# Frequencies of clinically important *CYP2C19* and *CYP2D6* alleles are graded across Europe

**DOI:** 10.1038/s41431-019-0480-8

**Published:** 2019-07-29

**Authors:** Jelena Petrović, Vesna Pešić, Volker M. Lauschke

**Affiliations:** 1 Department of Physiology and Pharmacology, Section of Pharmacogenetics, Karolinska Institutet, Stockholm, Sweden; 2 Faculty of Pharmacy, Department of Physiology, University of Belgrade, Belgrade, Serbia

**Keywords:** Genetic variation, Genetics research, Epidemiology

## Abstract

CYP2C19 and CYP2D6 are important drug-metabolizing enzymes that are involved in the metabolism of around 30% of all medications. Importantly, the corresponding genes are highly polymorphic and these genetic differences contribute to interindividual and interethnic differences in drug pharmacokinetics, response, and toxicity. In this study we systematically analyzed the frequency distribution of clinically relevant *CYP2C19* and *CYP2D6* alleles across Europe based on data from 82,791 healthy individuals extracted from 79 original publications and, for the first time, provide allele confidence intervals for the general population. We found that frequencies of *CYP2D6* gene duplications showed a clear South-East to North-West gradient ranging from <1% in Sweden and Denmark to 6% in Greece and Turkey. In contrast, an inverse distribution was observed for the loss-of-function alleles CYP2D6*4 and CYP2D6*5. Similarly, frequencies of the inactive CYP2C19*2 allele were graded from North-West to South-East Europe. In important contrast to previous work we found that the increased activity allele CYP2C19*17 was most prevalent in Central Europe (25–33%) with lower prevalence in Mediterranean-South Europeans (11–24%). In summary, we provide a detailed European map of common *CYP2C19* and *CYP2D6* variants and find that frequencies of the most clinically relevant alleles are geographically graded reflective of Europe’s migratory history. These findings emphasize the importance of generating pharmacogenomic data sets with high spatial resolution to improve precision public health across Europe.

## Introduction

Interindividual variability in therapeutic drug response can result in adverse drug reactions (ADRs) or lack of efficacy and constitutes a key challenge for health care systems. Notably, 40–70% of patients experience insufficient drug response or drug toxicity and ADRs account for 6.5% of all hospital admissions of which up to 30% are life threatening in at-risk subpopulations [1–4]. Genetic polymorphisms in drug-metabolizing enzymes, transporters, or drug targets explain around 20–30% to these interindividual differences [5].

Cytochrome P450 (CYP) enzymes constitute a polymorphic superfamily, consisting of 57 functional members in humans [6], that metabolize >80% of all clinically used medications [7]. Among those, *CYP2C19* and *CYP2D6* are of particular clinical relevance, as they are highly polymorphic and implicated in the metabolism of numerous widely prescribed drugs. CYP2C19 substrates include the tricyclic antidepressants amitriptyline, clomipramine, doxepin and imipramine, the selective serotonin reuptake inhibitors citalopram and sertraline, the antifungal voriconazole, as well as the antiplatelet agent clopidogrel. CYP2C19*2 (rs4244285) is the most common allelic variant in Caucasians and results in aberrant splicing and loss-of-enzyme activity [8]. In contrast, the regulatory polymorphism rs12248560 defining CYP2C19*17 increases transcriptional activity and causes the ultrarapid CYP2C19 metabolism [9].

CYP2D6 metabolizes around 25% of currently prescribed drugs, including various antidepressants, neuroleptics, beta-blockers, opioids, antiemetics, and antiarrhythmics. Of the more than 100 allelic variants for *CYP2D6* that have been described so far, CYP2D6*4 (rs3892097) is the most prevalent loss-of-function allele in Caucasian individuals. Furthermore, *CYP2D6* harbors functionally relevant copy number variations (CNVs) in which the whole open reading frame is duplicated (e.g., CYP2D6*1×N and CYP2D6*2×N) or deleted (CYP2D6*5), resulting in increased or decreased metabolism of CYP2D6 substrates, respectively.

While frequencies of *CYP2C19* and *CYP2D6* variations have been extensively studied, these studies were either focused on selected geographical regions or analyzed data aggregated by ethnicity or ancestry [10–12]. Therefore, in the present study, we systematically analyzed 79 original publications covering 82,791 healthy volunteers throughout Europe for *CYP2C19* and *CYP2D6* variants to provide a high-resolution map of pharmacogenetically relevant variability across European populations. Analysis of this consolidated data set revealed that the loss-of-function variants CYP2C19*2, CYP2D6*4, and CYP2D6*5 were graded from Northern Europe to the Mediterranean, whereas *CYP2D6* duplications showed an inverse pattern. Furthermore, in contrast to previous reports we find clear evidence that CYP2C19*17 is most common in Central Europe, whereas prevalence is lower in South Europeans. Combined, these  data reveal the extent of intra-European pharmacogenetic variability and underscore the importance of using local genomic information for conducting pharmacogenetic analyzes, clinical trials, and precision public health.

## Methods

For the present study we performed a systematic literature survey of the PubMed database covering articles published before December 2018. The search query criteria were (CYP2C19 or CYP2D6) AND (allele OR genotype OR frequency OR prevalence OR polymorphism) AND (European). All studies reporting genotype or allele frequencies of CYP2C19*2 (rs4244285; NC_000010.11:g.94781859 G > A), CYP2C19*17 (rs12248560; NC_000010.11:g.94761900C > T), CYP2D6*3 (rs35742686; NC_000022.11:g.42128242delT), CYP2D6*4 (rs3892097; NC_000022.11:g.42128945C > T), CYP2D6*5 (*CYP2D6* gene deletion), or of functional gene duplications (CYP2D6*1×N or CYP2D6*2×N) in healthy individuals of clear geographic origin within a European country were included. Variant positions are provided based on GRCh38. Only original research articles available in English were considered. In addition, we included data from the Genome Aggregation Database [13], the 1000 Genomes Project [14], the SweGen project [15], and the Estonian biobank [16]. As a result, we identified 79 original articles and 82,791 individuals were included in the analysis (Supplementary Tables 1 and 2). For countries for which multiple studies were available, data were aggregated using a weighted average approach using the studies’ cohort sizes as weighting factor. For additional information about the haplotypes in question we refer the interested reader to the website of the Pharmacogene Variation Consortium (https://www.pharmvar.org).

## Results

### Frequencies of important *CYP2C19* alleles exhibit large intra-European differences

**Fig. 1:**
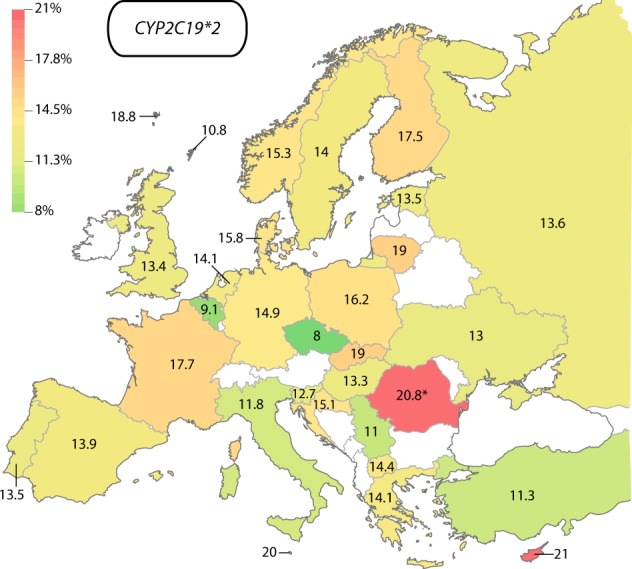
European map of CYP2C19*2 allele frequencies. The lowest frequencies were found in the Czech republic (8%, green), whereas highest frequencies were described in Cyprus (21%). Frequency in Romania (indicated by asterisk) refers exclusively to the Romani population

For *CYP2C19* we assessed the prevalence of the loss-of-function allele CYP2C19*2 and the increased function variant CYP2C19*17. In Europe, the frequency of CYP2C19*2 was the highest in Cyprus (21%) and Malta (20%), whereas the lowest prevalence was reported in Czech Republic (8%; Fig. 1; Table 1). Furthermore, frequencies were high in Romani individuals (20.8%). Overall, CYP2C19*2 was slightly more prevalent in Northern and Western European countries, such as Finland (17.5%), the Faroe Islands (18.8%), and France (17.7%), compared with countries on the Mediterranean coast, including Italy (11.8%) and Turkey (11.3%).

**Table 1:** Frequencies of important *CYP2C19* and *CYP2D6* alleles in Europe

Functional consequence			Frequency in %					
Country/Geographic region	Studies	Individuals	*CYP2C19*		*CYP2D6*			
			*2	*17	*3	*4	*5	Dupl
			Inactive	Increased function	Inactive	Inactive	Inactive	Increased function
Austria	1	93	/	/	0.5 (1.2)	14 (5.9)	1.6 (2.1)	1.6 (2.1)
Belgium	1	121	9.1 (4.3)	/	/	/	/	/
Croatia	3	1119–1247	15.2 (1.7)	23.5 (2)	2.3 (0.7)	16.7 (1.7)	1 (0.5)	3.4 (0.8)
Cyprus	1	40	21 (10.6)	11 (8.1)	4 (5.1)	21 (10.6)	/	/
Czech Republic	2	42–265	8 (2.7)	29 (4.6)	1.6 (1.3)	21.6 (4.2)	3.1 (1.8)	/
Denmark	3	579–634	15.8 (2.4)	20.1 (2.6)	2.2 (1)	20.5 (2.6)	5.9 (1.5)	0.8 (0.6)
Estonia	1	35,506–44,448	13.5 (0.3)	26.4 (0.3)	1.8 (0.1)	16.7 (0.3)	1.5 (0.1)	0.3 (0.04)
Faroe Islands	2	309–311	18.8 (3.6)	15.4 (3.4)	0.2 (0.4)	33.4 (4.4)	/	/
Finland	6	12,589–13,956	17.5 (0.5)	19.6 (0.6)	3.5 (0.3)	10 (0.4)	2.2 (0.2)	4.3 (0.3)
France	3	607	17.7 (2.6)	/	/	/	/	/
Germany	8	923–1758	14.9 (1.4)	24.9 (1.7)	1.1 (0.4)	19.6 (1.6)	3.2 (0.7)	1.3 (0.4)
Greece	3	327	14.1 (3.2)	18.2 (3.5)	2.1 (1.3)	17.7 (3.5)	/	6 (2.2)
Hungary	4	530–591	13.3 (2.3)	23 (2.9)	1.6 (0.9)	19 (2.7)	1.8 (0.9)	1.8 (0.9)
Italy	8	914–917	11.8 (1.8)	22.1 (2.3)	1 (0.5)	16.4 (2)	2.4 (0.8)	3 (0.9)
Lithuania	1	20	19 (14.4)	25 (15.9)	2 (5.2)	24 (15.7)	/	/
Malta	1	41	20 (10.3)	26 (11.3)	0	18 (9.9)	/	/
Netherlands	5	1114–1158	14.1 (1.7)	19 (1.9)	1.5 (0.6)	18.9 (1.9)	/	/
Norway	3	83–403	15.3 (3)	22 (3.4)	0	21.1 (3.3)	6 (2)	/
Orkney Islands	1	88	10.8 (5.4)	/	/	/	/	/
Poland	5	166–791	16.3 (2.2)	29.8 (2.7)	1.6 (0.7)	20.8 (2.4)	/	/
Portugal	4	279–400	13.4 (2.8)	/	0.7 (0.7)	17 (3.1)	2.6 (1.3)	3 (1.4)
Republic of North Macedonia	2	100–184	14.4 (4.3)	20.1 (4.9)	2 (1.7)	17 (4.6)	1.5 (1.5)	2.5 (1.9)
Romania (Romani)	3	426–562	20.8 (2.8)	/	/	22.5 (2.9)	/	/
Russia	4	391–1663	13.6 (1.4)	15 (1.4)	1.2 (0.4)	17.6 (1.5)	1.6 (0.5)	2.4 (0.6)
Sardinia	2	76	/	/	2.6 (3)	15.8 (6.9)	1.3 (2.1)	2 (2.6)
Serbia	1	46	11 (7.6)	18 (9.3)	0	16 (8.9)	/	/
Slovakia	1	26	19 (12.7)	33 (15.2)	2 (4.5)	28 (14.5)	/	/
Slovenia	3	1952–2081	12.7 (1.2)	23 (1.5)	1.8 (0.5)	16.8 (1.3)	/	/
Spain	14	1215–2328	14 (1.2)	17.1 (1.3)	1.2 (0.4)	18.6 (1.3)	2.3 (0.5)	3.5 (0.6)
Sweden	6	1816–2020	14 (1.3)	19.2 (1.4)	1.6 (0.5)	20.7 (1.5)	4.1 (0.7)	0.5 (0.3)
Turkey	6	689–785	11.3 (1.9)	24 (2.5)	0.7 (0.5)	13.2 (2)	1.8 (0.8)	5.6 (1.4)
Ukraine	2	52–689	13 (2.1)	25 (2.7)	2 (0.9)	18.9 (2.5)	/	/
United Kingdom	2	91–168	13.4 (4.3)	24.2 (5.4)	3.3 (2.3)	24.2 (5.4)	/	/

Note that the number of individuals in a given country or geographic region for whom genotype data are available can differ between alleles. Values in brackets indicate the 90% confidence intervals

**Fig. 2:**
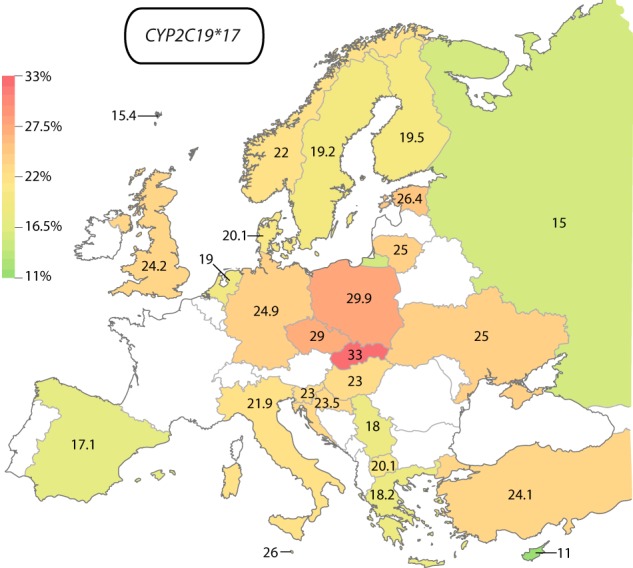
European map of CYP2C19*17 allele frequencies. The lowest frequencies were found in Cyprus (11%, green), whereas highest frequencies were described in Slovakia (33%)

On the contrary, CYP2C19*17 was most common in Central Europe with highest frequencies in Slovakia (33%), Poland (29.8%), and the Czech Republic (29%; Fig. 2); Table 1. However, the *CYP2C19* genotyping data reported for Slovakia included only 26 subjects and should thus be interpreted with caution [17]. In contrast, frequencies were lower in Southern European countries, such as Spain (17.1%), Greece (18.2%), and Cyprus (11%), as well as Scandinavia (19–22%) and Russia (15%).

### *CYP2D6* gene duplications are graded from South-East to North-West Europe

**Fig. 3:**
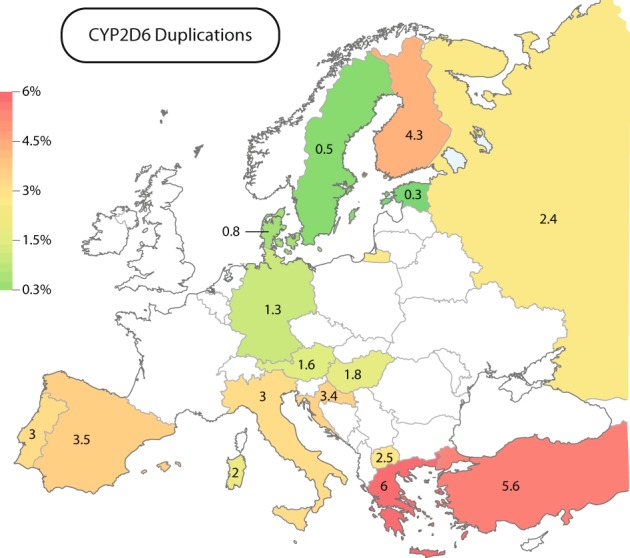
European map of *CYP2D6* allele duplications (CYP2D6*1×N and CYP2D6*2×N). The lowest frequencies were found in Northern European countries, such as Estonia (0.3%) and Sweden (0, 5%, green), whereas highest frequencies were described in South-Eastern Europe (Greece; 6% and Turkey; 5.6%, red)

Functional duplications  of *CYP2D6* (CYP2D6*1×N and CYP2D6*2×N) were most prevalent in the South-East European countries Greece (6%) and Turkey (5.6%), while lower frequencies were found in South-Western Europe, including Spain (3.5%), Italy (3%), and Portugal (3%; Fig. 3; Table 1). In contrast, frequencies in Northern and Central Europe, including Austria (1.6%), Germany (1.3%), Denmark (0.8%), and Sweden (0.5%), were substantially lower. Surprisingly, *CYP2D6* duplications were common in Finland (4.3%) at levels similar to Southern Europe.

### *CYP2D6* loss-of-function alleles are distributed along a North-to-South gradient

**Fig. 4:**
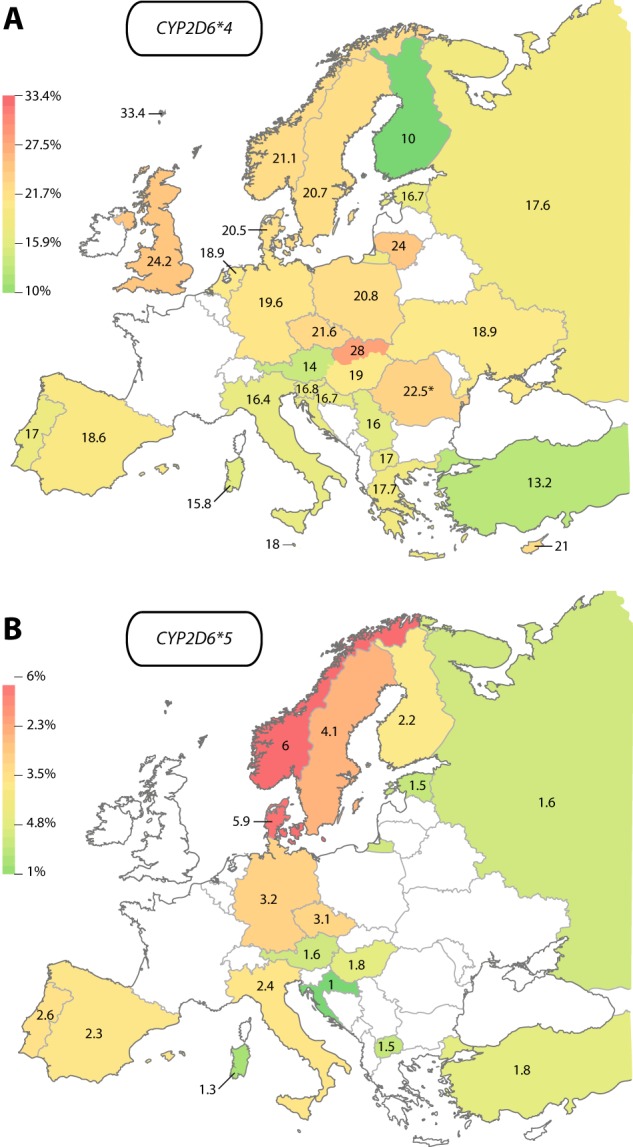
European maps of the *CYP2D6* loss-of-function alleles CYP2D6*4 and CYP2D6*5. **a** CYP2D6*4 frequencies differed between 10% in Finland (green) and 33.4% on the Faroe Islands (red). Frequency in Romania (indicated by asterisk) refers exclusively to the Romani population. **b** CYP2D6*5 was most common in Norway (6%, red) and Denmark (5.9%), whereas it was most rare in Croatia (1%)

Importantly, the *CYP2D6* loss-of-function alleles CYP2D6*4 and CYP2D6*5 showed an inverse profile (Fig. 4; Table 1). CYP2D6*4 was most prevalent throughout Northern and Central Europe with frequencies pivoting around 20–25%. The highest CYP2D6*4 frequency in Europe was observed on the Faroe Islands (33.4%). In contrast, frequencies were substantially lower in most Southern European countries, such as Turkey (13.2%), Italy (16.4%), and Greece (17.7%). Notably, Finns contradict this trend with a population frequency of 10%, which is substantially lower than in neighboring Sweden (19.2%), Norway (22%), and Estonia (16.7%).

Similar trends were observed for the *CYP2D6* deletion variant CYP2D6*5, which was most frequent in Norway (6%), Denmark (5.9%), and Sweden (4.1%), whereas prevalence in Central Europe pivoted around 3% and lowest CYP2D6*5 frequencies were observed in Southern European countries, such as Croatia (1%), Sardinia (1.3%), North Macedonia (1.5%), and Turkey (1.8%). Again, population frequency of CYP2D6*5 in Finland (2.2%) contrasted surrounding Scandinavian countries and was more similar to prevalence rates in Central Europe.

In contrast to CYP2D6*4 and CYP2D6*5, no clear gradients were detected for CYP2D6*3, whose frequencies pivoted around 0–2% throughout Europe. Notable exceptions are the relatively high, geographically disperse frequencies in Cyprus (4%), Finland (3.5%), and the UK (3.3%; Supplementary Fig. 1).

## Discussion

Interethnic differences in drug pharmacokinetics or dynamics constitute important factors to consider for increasingly multinational drug development programs and genetic variability in drug-metabolizing enzymes constitutes an important factor underlying these differences. As a result, the labels of multiple marketed drugs, including rosuvastatin, carbamazepine, and tacrolimus, contain recommendations to adjust starting doses based on ethnicity [18]. *CYP2C19* and *CYP2D6* harbor multiple genetic polymorphisms, which differ substantially between ethnic groups and geographic regions and can entail clinically important differences in drug response. To date, numerous studies have analyzed the frequencies of these polymorphisms; yet, the available allele frequency data have, to our knowledge, not yet been systematically consolidated into high-resolution maps of *CYP2C19* and *CYP2D6* variability within Europe. We therefore compiled data from 79 original publications resulting in aggregated genotypes for the most relevant *CYP2C19* and *CYP2D6* alleles from 82,791 healthy individuals. Notably, while most studies provided data from unrelated individuals, we cannot exclude relatedness across studies. However, we do not expect this fraction to significantly impact the accuraccy of  the reported frequencies.

Frequency of functional *CYP2D6* gene duplications was highest in Greece and Turkey and lowest in Scandinavian countries, which is in accordance with decreasing frequencies of ultrarapid metabolizers in a direction from Southern to Northern European populations [19]. Globally, *CYP2D6* duplication is most common in North-East Africa and the Middle East with frequencies of 7–16% [20–22]. It has been speculated that the evolutionary basis for this gradient is the role of CYP2D6 in the detoxification of plant alkaloids, which allowed carriers of duplicated alleles to tap food sources during times of starvation that would have been toxic for normal CYP2D6 metabolizers [23]. Inversely, frequencies of the loss-of-function alleles CYP2D6*4 and CYP2D6*5 were highest in Scandinavia and lowest on the Mediterranean with further decreasing frequencies in Ethiopia and the Arabian peninsula [20–22]. These data thus corroborate the hypothesis that CYP2D6 metabolic capacity might have been under selective pressure specifically in North-East Africa and subsequent migration events resulted in the high frequencies of ultrarapid CYP2D6 metabolizers in Southern Europe.

We observed that CYP2C19*2 was graded from North-West to South-East Europe. Interestingly, we observed a high frequency of CYP2C19*2 in Romani (20.8%) that was significantly different from the hosting Hungarian population (13.3%; *p* < 0.01; [24]). The Roma minority originates from North-West India, and due to a series of population bottlenecks with multiple founder events and low number of interethnic marriages constitutes a relatively homogeneous ethnic group [25]. As a consequence of this complex population history, CYP2C19*2 frequencies in Roma were similar to those reported in North Indian populations [26]. Thus, pharmacogenetic variability in Roma is distinctly different from European populations and affiliation to a Roma group might be a factor of consideration for treatment decisions of CYP2C19 substrates.

The distribution of CYP2C19*17 was highest in Central Europe and lower in Southern European countries. Our findings are in drastic contrast to a meta-analysis performed by Fricke-Galindo et al. who reported that CYP2C19*17 is predominantly found in Mediterranean countries with frequencies of 42% [11]. However, we find that frequencies are substantially lower throughout Southern Europe, pivoting around 20–25%. Careful revisiting of the original data revealed that instead of the frequency (14.9%), Fricke-Galindo et al. erroneously used the number of individuals (*n* = 42) for the Spanish population [27] as population frequency. Our findings of moderate CYP2C19*17 frequencies in Southern Europe align with data from Northern African and Middle Eastern populations in which CYP2C19*17 allele frequencies between 17.9% and 26.9% have been reported for Ethiopians, Saudi Arabians, Kurds, and Turks [9, 28–30]. Furthermore, low frequencies (15.9%) have been found in Sephardic Jews [31] who originated from Jews on the Iberian peninsula in the 15th century, which are in close agreement with the aggregated prevalence we found in contemporary Spanish individuals (17.1%). The distribution of *CYP2C19* alleles thus reflects the migratory history of European populations.

These findings have potentially important implications, as *CYP2C19* genotype is included as a pharmacogenomic biomarker in the drug labels of 22 medications. Furthermore, guidelines issued by pharmacogenetics expert workgroups (CPIC and DPWG) provide recommendations to optimize genotype-guided prescription for 14 drugs [32]. For instance, *CYP2C19* genotype affects treatment efficacy and risk of adverse events when treated with the antidepressant escitalopram [33], and for ultrarapid CYP2C19 metabolizers it is recommended to select an alternative drug not predominantly metabolized by CYP2C19. As the cost effectiveness of pharmacogenetic implementation is dependent on carrier frequencies, falsely high population frequencies might erroneously incentivize pre-emptive *CYP2C19* genotyping.

Notably, while genotype data for *CYP2C19* and *CYP2D6* were available for more than 80,000 individuals from 31 European countries, cohort coverage was geographically highly unequal (Table 1). For eight countries less than 100 individuals were genotyped and, as a result, population frequencies in these countries could only be estimated with wide confidence intervals. Thus, these analyzes incentivize the country-specific expansion of genotype data to further refine estimates of intra-European *CYP* allele frequencies. Furthermore, while *CYP* genotype-derived activity scores constitute important proxies for the prediction of metabolic capacity, they can only explain a fraction of the observed functional variability [34]. One underlying reason could be rare variants beyond the tested polymorphisms that contribute to gene function. In this regard *CYP2C19* and *CYP2D6* have indeed been found to harbor a plethora of rare genetic single nucleotide variants (SNVs) with putative functional importance [35–37]. Furthermore, rare population-specific CNVs can contribute to functional variability. For instance, *CYP2C19* has recently been found to be deleted specifically in Finns with frequencies of 0.8% [38]. However, information regarding the prevalence of these rare SNVs and CNVs is currently not available with high geographic resolution and the generation of such sequencing-based pharmacogenomic data sets constitutes an interesting avenue for future research that will help to refine genotype-guided drug response predictions [39, 40].

In conclusion, we provide refined maps of clinically important *CYP2C19* and *CYP2D6* genetic variability across European populations. Our findings support the need for refined pharmacogenomic mapping to guide precision public health.

## Supplementary information

Supplementary Table 1

Supplementary Table 2

Supplementary Figure 1

Supplementary Material
