# Supplementary figures and images for: Frequencies of clinically important CYP2C19 and CYP2D6 alleles are graded across Europe
Source: Eur J Hum Genet. 2019 Jul 29;28(1):88–94. doi: 10.1038/s41431-019-0480-8 (PMC6906321; doi:10.1038/s41431-019-0480-8)

# Supplementary Figure 1

*CYP2D6\*3*

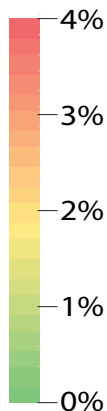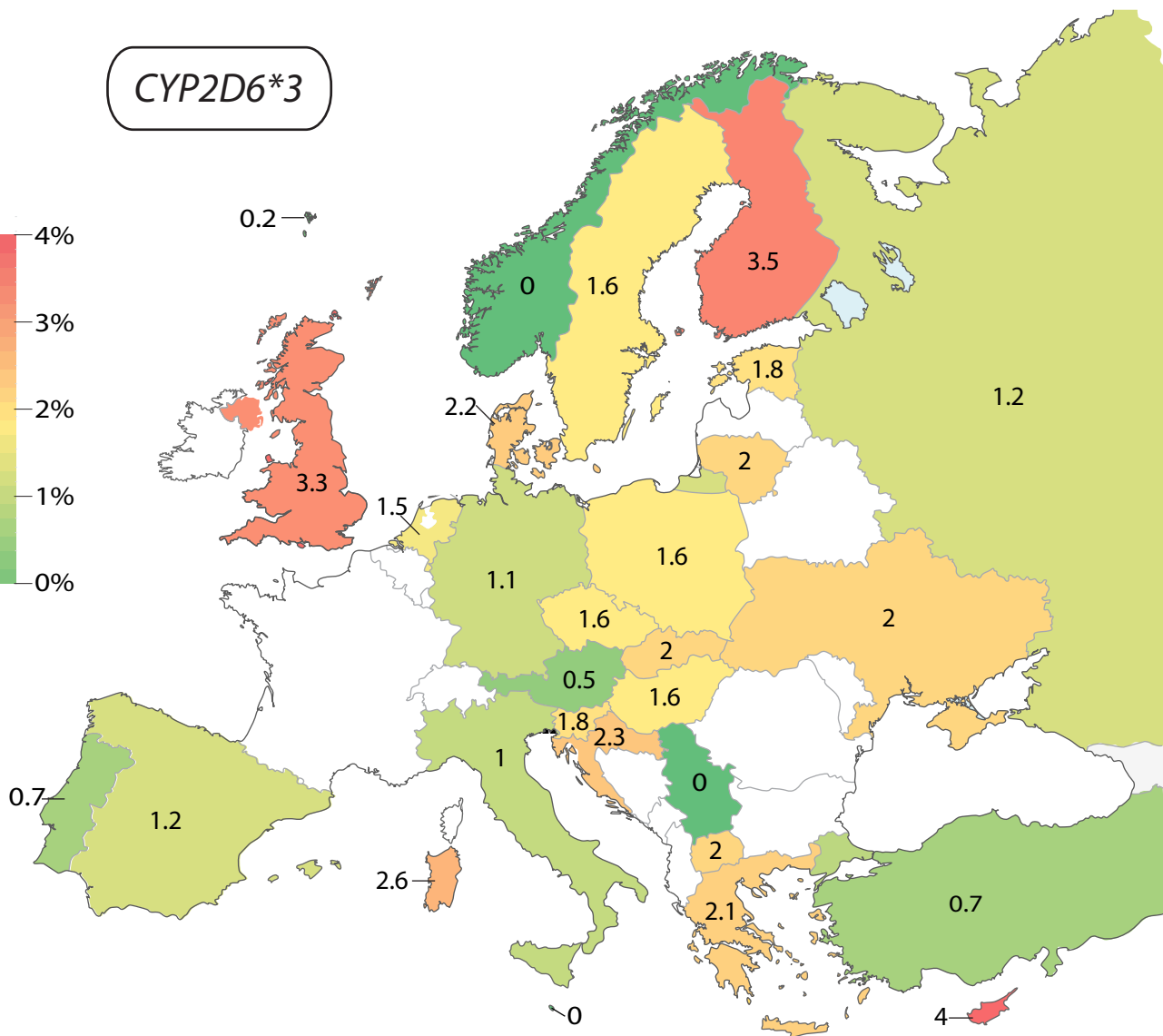

Supplement: Supplementary file 3 — Supplementary Figure 1 [file 41431_2019_480_MOESM3_ESM.pdf]
