# Supplementary material for: Frequencies of clinically important CYP2C19 and CYP2D6 alleles are graded across Europe
Source: Eur J Hum Genet. 2019 Jul 29;28(1):88–94. doi: 10.1038/s41431-019-0480-8 (PMC6906321; doi:10.1038/s41431-019-0480-8)
Supplement: Supplementary file 4 — Supplementary Material [file 41431_2019_480_MOESM4_ESM.docx]

**Supplementary Material**

**Supplementary Tables: 2**

**Supplementary Figures: 1**

**Supplementary Figure 1: European map of *CYP2D6*3* allele frequencies.** *CYP2D6*3* was not identified in individuals in Norway, Malta and Serbia (green) whereas highest frequencies were found in Cyprus (4%).
